# Genome-Wide Association Analysis of Eating Disorder-Related Symptoms, Behaviors, and Personality Traits

**DOI:** 10.1002/ajmg.b.32087

**Published:** 2012-08-22

**Authors:** Vesna Boraska, Oliver SP Davis, Lynn F Cherkas, Sietske G Helder, Juliette Harris, Isabel Krug, Thomas Pei-Chi Liao, Janet Treasure, Ioanna Ntalla, Leila Karhunen, Anna Keski-Rahkonen, Danai Christakopoulou, Anu Raevuori, So-Youn Shin, George V Dedoussis, Jaakko Kaprio, Nicole Soranzo, Tim D Spector, David A Collier, Eleftheria Zeggini

**Affiliations:** 1 Wellcome Trust Sanger Institute, Wellcome Trust Genome CampusHinxton, Cambridge, UK; 2 Department of Medical Biology, University of Split School of MedicineSplit, Croatia; 3 MRC Social Genetic and Developmental Psychiatry Centre, Institute of Psychiatry, King's College LondonLondon, UK; 4 Department of Twin Research & Genetic Epidemiology, King's College London, St Thomas' Hospital CampusWestminster Bridge Road, London, UK; 5 School of Psychological Sciences, University of MelbourneMelbourne, Victoria, Australia; 6 Department Academic Psychiatry, King's College LondonLondon, UK; 7 Department of Dietetics and Nutrition, Harokopio University of AthensAthens, Greece; 8 Department of Clinical Nutrition, Institute of Public Health and Clinical Nutrition, University of Eastern FinlandKuopio, Finland; 9 Department of Public Health, The Hjelt Institute, University of HelsinkiHelsinki, Finland; 10 Institute of Molecular Medicine, University of HelsinkiHelsinki, Finland; 11 Unit for Child and Adolescent Mental Health, National Institute for Health and WelfareHelsinki, Finland

**Keywords:** Drive for Thinness, Body Dissatisfaction, Childhood Obsessive Compulsive Personality Disorder, Weight Fluctuation, Breakfast Skipping

## Abstract

Eating disorders (EDs) are common, complex psychiatric disorders thought to be caused by both genetic and environmental factors. They share many symptoms, behaviors, and personality traits, which may have overlapping heritability. The aim of the present study is to perform a genome-wide association scan (GWAS) of six ED phenotypes comprising three symptom traits from the Eating Disorders Inventory 2 [Drive for Thinness (DT), Body Dissatisfaction (BD), and Bulimia], Weight Fluctuation symptom, Breakfast Skipping behavior and Childhood Obsessive-Compulsive Personality Disorder trait (CHIRP). Investigated traits were derived from standardized self-report questionnaires completed by the TwinsUK population-based cohort. We tested 283,744 directly typed SNPs across six phenotypes of interest in the TwinsUK discovery dataset and followed-up signals from various strata using a two-stage replication strategy in two independent cohorts of European ancestry. We meta-analyzed a total of 2,698 individuals for DT, 2,680 for BD, 2,789 (821 cases/1,968 controls) for Bulimia, 1,360 (633 cases/727 controls) for Childhood Obsessive-Compulsive Personality Disorder trait, 2,773 (761 cases/2,012 controls) for Breakfast Skipping, and 2,967 (798 cases/2,169 controls) for Weight Fluctuation symptom. In this GWAS analysis of six ED-related phenotypes, we detected association of eight genetic variants with *P* < 10^−5^. Genetic variants that showed suggestive evidence of association were previously associated with several psychiatric disorders and ED-related phenotypes. Our study indicates that larger-scale collaborative studies will be needed to achieve the necessary power to detect loci underlying ED-related traits. © 2012 Wiley Periodicals, Inc.

## INTRODUCTION

Eating disorders (EDs) are complex psychiatric disorders involving both genetic and environmental factors. They cover a variety of psychiatric diagnoses, with the most common being anorexia nervosa (AN), bulimia nervosa (BN), and eating disorders not otherwise specified (EDNOS) [Treasure et al., 2010]. Family and twin studies have shown that AN and BN are heritable and aggregate in first-degree relatives of patients [Walters and Kendler, 1995; Lilenfeld et al., 1998; Strober et al., 2000]. However, EDs are not likely to be etiologically separate from each other, as patients can move from one diagnosis to another over time (e.g., from AN to BN), they share many symptoms and environmental risk factors, and have overlapping heritability, most likely from shared genetic susceptibility loci [Strober et al., 2000; Wade et al., 2000; Helder and Collier, 2011].

Many candidate genes, such as *BDNF* and *HTR2A*, have been examined for association with EDs, mainly for AN [Pinheiro et al., 2010; Rask-Andersen et al., 2010]. Several linkage studies of AN and BN have been performed for both clinical diagnosis and ED-related symptoms, including AN and BN as categorical diagnosis, as well as symptoms scores from the Eating Disorders Inventory (EDI2)—Drive for Thinness (DT), Body Dissatisfaction (BD) and Bulimia, and other symptom measures such as obsessionality, perfectionism, and food-related obsessions. These studies implicated several loci for AN [Devlin et al., 2002; Grice et al., 2002], BN [Bulik et al., 2003], obsessionality [Devlin et al., 2002; Bacanu et al., 2005], food-related obsessions [Bacanu et al., 2005], and DT [Devlin et al., 2002]. In addition, two genome-wide association studies have been performed, one using microsatellite markers [Nakabayashi et al., 2009], and one using SNPs [Wang et al., 2011]. However, genome-wide significance of these findings has not yet been achieved, partly because of the paucity of available datasets of sufficient sample size (and hence power) for replication studies.

The present study examined ED-related symptoms, behaviors and traits, derived from standardized self-report instruments. These were the DT, BD, and Bulimia traits from the EDI-2, a self-report questionnaire used to assess the presence of EDs which has also been widely used as a population quantitative trait measure. In addition, we analyzed Weight Fluctuation (WF), another feature of ED pathology, which was assessed by the Hermann and Polivy Restraint Scale questionnaire. We also included a question on Breakfast Skipping and a self-report measure of Childhood Obsessive-Compulsive Personality Disorder (OCPD) traits (CHIRP), which are both risk factors for the development of EDs. The study of these ED-related phenotypes, as potential intermediate phenotypes, may be useful in identifying genetic variation underlying EDs and detecting the biological mechanisms contributing and modifying them [Gottesman and Gould, 2003; Morris et al., 2010].

ED-related symptoms such as DT and BD are also associated with depressive symptoms and psychological distress, which are common among ED patients [Lautenbacher et al., 1992; McCabe and Marwit, 1993; Keski-Rahkonen et al., 2005]. The heritability of DT, BD, and Bulimia has been assessed by twin model-fitting in the TwinsUK data and yielded heritability estimates of 0.57 for DT, 0.65 for BD and 0.54 for Bulimia. Genetic correlations among these traits are 0.69 on average (O.S.P. Davis et al., unpublished data). These estimates agree closely with estimates from other twin samples [Keski-Rahkonen et al., 2005]. Another ED-related symptom, WF, is linked to cycles of dieting and overeating and is also implicated as a genetic marker in EDs [Heatherton et al., 1991].

An important trait related to EDs is Breakfast Skipping. This is associated with poor health behavior, including behavioral disinhibition and high body mass index [Keski-Rahkonen et al., 2003]. The literature has shown that women with subclinical EDs skip breakfast more often than healthy controls suggesting that breakfast skipping may be associated with milder disordered eating patterns [Melve and Baerheim, 1994; Keski-Rahkonen et al., 2004]. The heritability of Breakfast Skipping has been assessed by twin modeling studies suggesting that additive genetic effects explain 0.41 of the variance in girls and 0.66 in boys [Keski-Rahkonen et al., 2004].

Individuals with EDs also share many personality and temperament features such as obsessive-compulsive traits, perfectionism, and harm avoidance [Casper, 1990; Fassino et al., 2004; Halmi et al., 2005]. Many of these personality characteristics are elevated in unaffected family members of individuals with EDs, also exist premorbidly, and may confer risk to the development of EDs [Casper, 1990; Bulik et al., 2007]. OCPD traits, for example, are important risk factors in the development of EDs [Fairburn et al., 1999; Anderluh et al., 2003]. OCPD traits are also clinically important since they commonly persist from childhood into adulthood among AN patients [Altman and Shankman, 2009]. The heritability of OCPD traits, using the CHIRP questionnaire [Southgate et al., 2008] has been examined in the TwinsUK dataset and it is estimated that heritability accounts for 0.81 of the genetic variance and non-shared environmental factors account for the remaining 0.19 (T. Liao et al., unpublished data).

The aim of the present study is to carry out a genome-wide association scan (GWAS) of six ED-related phenotypes consisting of four ED-related symptom measures, Breakfast Skipping behavior and OCPD traits in a population-based twin cohort of British origin and a follow-up of interesting signals in two independent cohorts of European origin.

## MATERIALS AND METHODS

### Discovery Dataset—TwinsUK

The four ED-related symptoms, Breakfast Skipping and the OCPD traits were measured by self-completion of questionnaires that were sent to female twins from the UK Adult Twin Registry based at the Twin Research Unit at St. Thomas Hospital, London (http://www.twinsuk.ac.uk). More information on subjects, genome-wide genotyping and quality control (QC) procedures of the TwinsUK dataset can be found in Supplementary Material.

### Self-Report Questionnaires in TwinsUK

The six ED phenotypes were derived from standardized self-report instruments. Four ED-related symptoms were measured by the EDI-2 self-report assessment consisting of three subscales that assess DT, BD, and Bulimia, and the Herman-Polivy Restraint Scale questionnaire measuring WF. A five-point Likert scale assessed Breakfast Skipping. For the OCPD traits, the Childhood Retrospective Perfectionism Questionnaire (CHIRP) was used. 1/squared root transformation was applied to normalize the distribution of DT scores and was analyzed together with BD as a quantitative phenotype, whereas Bulimia, WF, Breakfast Skipping, and OCPD phenotypes were dichotomized because their distribution could not be transformed to normality. Detailed information on standardized self-report instruments can be found in Supplementary Material. The sample size and clinical characteristics of TwinsUK individuals are shown in Table I.

**TABLE I:** Number of Samples with Genotype and Phenotype Data in the Discovery TwinsUK Dataset

Phenotype	Type	Samples (n), case/control	Phenotype, mean (SD); % cases (for bn)	Age, years, mean (SD)	BMI, kg/m^2^, mean (SD)
TwinsUK					
DT	qt	1,934	17.42 (6.96)	58.20 (11.90)	25.13 (4.67)
BD	qt	1,915	27.35 (9.98)	58.07 (11.94)	25.11 (4.67)
BULIMIA	bn	531/1,493	26.24	58.26 (11.91)	25.13 (4.66)
OCPD	bn	257/626	29.11	57.99 (12.33)	25.21 (4.83)
BREAKFAST SKIPPING	bn	403/1,603	20.09	60.08 (11.88)	25.16 (4.65)
WF	bn	678/1,889	26.41	56.48 (13.04)	25.31 (4.89)

Phenotypes: DT, Drive for Thinness; BD, Body Dissatisfaction; OCPD, Childhood Obsessive Compulsive Personality Disorder trait; WF, Weight Fluctuation; qt, quantitative trait; bn, binary trait.

### Association Analysis in TwinsUK

We tested 283,744 directly typed overlapping SNPs for association with the six ED phenotypes of interest. Association analyses were restricted to individuals with phenotype information: 1,934 individuals for DT, 1,915 for BD, 2,024 for Bulimia, 883 for OCPD, 2,006 for Breakfast Skipping, and 2,567 for WF (Table I). All analyses were adjusted for family relatedness. The genomic control (GC) value was estimated to be 1.002. Analyses have not been adjusted for age and sex. All TwinsUK individuals consisted of females only. We created QQ and Manhattan plots to visualize association analysis results. Detailed information on association analysis can be found in Supplementary Material.

### Estimating the Total Variance Accounted for by Tagged SNPs in TwinsUK

We estimated the proportion of the phenotypic variation accounted for by the 283,744 overlapping SNPs in the TwinsUK sample using the GCTA software (http://gump.qimr.edu.au/gcta/). Analyses details can be found in Supplementary Material.

### *In Silico* Replication—FinnTwin16

#### Prioritization of SNPs for in silico replication

On the basis of the discovery dataset results we prioritized 27 SNPs across all six traits of interest: we focused on SNPs with association *P*-values below or close to 10^−5^ and additionally examined the genomic location of the most strongly associated SNPs, prioritizing those within or near biologically interesting genes. We visually inspected cluster plots for all selected SNPs prior to replication. Association summary statistics for the 27 SNPs that were taken forward to replication are presented in Supplementary Table I. The study design is presented in Supplementary Figure 1.

#### In silico replication samples

Subjects were ascertained from a population-based Finnish twin cohort study, FinnTwin16, based at the University of Helsinki. Detailed information on subjects, phenotypic correlation between quantitative phenotypes, genome-wide genotyping, and QC procedures for the FinnTwin16 dataset can be found in Supplementary Material. The analyzed dataset included 291 (167 males/124 females) individuals who had completed four ED-related questionnaires (DT, BD, Bulimia, and the Breakfast Skipping item). All ED-related questionnaires were identical to the self-completion questionnaires measured in the TwinsUK dataset. The sample size and clinical characteristics of FinnTwin16 individuals are presented in Table II. Eighteen out of 27 initially prioritized SNPs were examined in the FinnTwin16 dataset due to the fact that this cohort had phenotype measures for four out of the six ED-related variables.

**TABLE II:** Number of Samples With Genotype and Phenotype Data Across Replication Studies

Phenotype	Type	Samples (n), case/control	Phenotype, mean (SD); % cases (for bn)	Samples (n), case/control	Phenotype, mean (SD); % cases (for bn)
	FinnTwin16			TEENAGE	
DT	qt	284	15.56 (7.06)	480	19.71 (8.90)
BD	qt	285	21.61 (11.05)	480	20.39 (9.81)
BULIMIA	bn	80/205	28.07	210/270	43.76
OCPD	bn	/	/	376/101	78.8
BREAKFAST SKIPPING	bn	159/128	55.4	199/281	41.46
WF	bn	/	/	120/280	30
Age, years, mean (SD)		24.96 (1.41)		15.27 (1.03)	
BMI, kg/m^2^ mean (SD)	22.24 (3.48)			21.51 (3.42)	

Phenotypes: DT, Drive for Thinness; BD, Body Dissatisfaction; OCPD, Childhood Obsessive Compulsive Personality Disorder trait; WF, Weight Fluctuation; qt, quantitative trait; bn, binary trait.

#### Association analysis in FinnTwin16

We obtained raw genotype data for 18 directly typed SNPs and tested them for association with three ED-related symptoms (DT, BD, Bulimia) and the Breakfast Skipping item. Analyses were performed in the same way as in the TwinsUK dataset.

#### Prioritization of SNPs for de novo replication

We performed a meta-analysis of 18 SNPs across TwinsUK and FinnTwin16 to get a better estimate of significance and selected a subset of SNPs for further follow-up. Fifteen SNPs were taken forward for *de novo* genotyping in the TEENAGE cohort on the basis of these meta-analysis results. Also, nine SNPs, already selected for replication for the OCPD and WF phenotypes on the basis of discovery dataset results (variables not phenotyped in the FinnTwin16) were included in the list of SNPs for de novo replication.

#### EDs/obesity-related SNPs

In the TwinsUK dataset, we also examined 57 SNPs that had previously been associated with obesity or showed some evidence of association with EDs. We examined proxy SNPs (based on HapMap phase II), when the originally associated SNP was not directly typed. Six of these SNPs had *P* < 0.01 in at least one of the six analyses carried out here and were included for *de novo* replication: rs10501087 (*BDNF*), rs2294239 (*ZNRF3-KREMEN1*), and rs4846565 (*LYPLAL1*)—previously associated with obesity and rs6265 (*BDNF*), rs6604568 (near *SPATA17*), and rs1042114 (*OPRD1*)—previously associated with EDs. In total, 30 SNPs were taken forward for *de novo* genotyping in the TEENAGE cohort.

### *De Novo* Replication—TEENAGE

#### De novo replication samples and association analysis

The TEENs of Attica: Genes & Environment (TEENAGE) target population consisted of adolescent students attending all three classes of public secondary schools in the Attica region of Greece. ED-related symptoms, the Breakfast Skipping item and the OCPD traits were measured by self-report questionnaires. A total of 480 unrelated subjects (216 males/264 females) of the TEENAGE cohort were included in the present study. The sample size and clinical characteristics of TEENAGE individuals are presented in Table II. All six ED-related instruments were identical to self-completion assessments in TwinsUK. All 30 prioritized SNPs passed QC criteria. An additive linear or logistic, where appropriate, regression model was applied for association analysis. Detailed information on subjects, phenotyping, de novo genotyping and QC procedures in the TEENAGE cohort can be found in Supplementary Material.

#### Correlation between DT and BD quantitative phenotypes in three investigated datasets

Correlation between DT and BD phenotypes was calculated using Stata (Version 11.0; StataCorp, College Station, TZ).

### Meta-Analysis Across the TwinsUK, FinnTwin16, and TEENAGE Datasets

To assess the overall significance of prioritized SNPs we performed meta-analyses across the discovery and replication datasets, comprising 2,698 individuals for DT, 2,680 for BD, 2,789 (821 cases/1,968 controls) for Bulimia, 1,360 (633 cases/727 controls) for OCPD, 2,773 (761 cases/2,012 controls) for Breakfast Skipping, and 2,967 (798 cases/2,169 controls) for WF (Tables I and II). For three ED-related symptoms (DT, BD, Bulimia) and the Breakfast Skipping item, meta-analyses were conducted across all three datasets, whereas for the two remaining variables, OCPD traits and WF, meta-analyses were conducted across the TwinsUK and TEENAGE datasets. In addition, six SNPs previously associated with EDs and obesity were meta-analyzed across the TwinsUK and TEENAGE datasets only. We calculated the power of the study and investigated evidence for heterogeneity using I^2^ statistics. The genome-wide significance threshold was set to 5 × 10^−8^.

## RESULTS

No genome-wide significant associations were observed in the initial analysis of the discovery TwinsUK dataset. QQ plots for the six ED phenotypes are shown in Supplementary Figure 2. An estimate of the total variance accounted for by tagged SNPs per phenotype is shown in Supplementary Table II. Correlation values between DT and BD quantitative phenotypes in three investigated datasets are: r^2^ = 0.614 (TwinsUK), r^2^ = 0.735 (FinnTwin16), and r^2^ = 0.66 (TEENAGE). Of the 18 SNPs taken forward to *in silico* replication in FinnTwin16, none showed association with *P* < 0.05. Eleven of 18 signals had effects in the same direction as in the discovery dataset (binomial *P*-value = 0.48, given we expect 50% of signals to be in the same direction by chance; Supplementary Table I). Of the 24 SNPs prioritized for *de novo* replication in TEENAGE, two SNPs achieved *P* < 0.05. Eleven SNPs had effects in the same direction as in the discovery dataset (binomial *P*-value = 0.839; Supplementary Table I).

### Meta-Analysis Across the TwinsUK, FinnTwin16, and TEENAGE Datasets

In total, 27 SNPs were prioritized on the basis of the discovery GWAS results and followed up in two cohorts: 18 SNPs in FinnTwin16 and 24 SNPs in TEENAGE, 15 of which overlapped between both replication datasets. Meta-analysis across three datasets resulted in no genome-wide significant associations (Supplementary Table I). Eight SNPs reached *P* < 1 × 10^−5^ (Table III). Evidence for association for five SNPs improved in the meta-analysis, when compared to the discovery dataset (Table III). Meta-analysis had 80% power to detect an allele with frequency 0.35 and effect size of beta > 0.01 for DT, beta = 1.9 for BD, OR = 1.45 for Bulimia, OR = 1.65 for OCPD, OR = 1.5 for Breakfast Skipping, and OR = 1.5 for WF at the genome-wide significance level (*P* = 5 × 10^−8^).

**TABLE III:** Meta-Analysis Results of SNPs With *P* < 1 × 10^−5^

SNP information				Global meta-analysis								
	
MARKER	CHR	POS	NEAREST GENE	EA	EAF	BETA	SE	OR	95% CI	*P*	I^2^	N
BD												
**rs6894268**	5	178965094	*RUFY1*	A	0.354	1.439	0.305	/	/	2.38E−06	0	2,669
BULIMIA												
**rs7624327**	3	158319600	*CCNL1*	A	0.098	0.107	0.023	1.13	1.06–1.16	3.34E−06	0.437	2,784
OCPD												
rs7690467	4	133545845	No gene in 500 kb	C	0.285	−0.113	0.025	0.893	0.85–0.938	6.53E−06	0	1,360
rs1898111	15	45679590	*SEMA6D*	G	0.163	−0.137	0.031	0.872	0.82–0.93	7.66E−06	0.78	1,359
**rs10519201**	15	47008883	SHC4	T	0.132	0.147	0.032	1.158	1.09–1.23	6.16E−06	0	1,359
rs1557305	18	4711359	*DLGAP1*	T	0.372	−0.104	0.023	0.901	0.86–0.94	4.66E−06	0.65	1,358
WF												
**rs4853643**	2	192400015	*SDPR*	T	0.178	−0.074	0.017	0.929	0.89–0.96	9.88E−06	0	2,961
**rs218361**	8	116179901	*TRPS1*	G	0.429	0.059	0.013	1.061	1.03–1.08	7.01E−06	0	2,966

CHR, chromosome; POS, position; EA, effect allele; EAF, effect allele frequency; SE, standard error; OR, odds ratio; CI, 95% confidence intervals; *P*, *P*-value; I^2^, measure of heterogeneity; N, total number of samples; five SNPs shown in bold improved their association *P*-values in the meta-analysis.

### Established EDs and Obesity Loci

We examined 14 SNPs implicated by candidate and GWAS studies with EDs for the six ED phenotypes of interest in the TwinsUK discovery dataset (Supplementary Table III). Three SNPs showed association with *P* < 0.01: rs6265 (*BDNF*), rs6604568 (near *SPATA17*), and rs1042114 (*OPRD1*). We also examined 43 SNPs previously associated with obesity in the TwinsUK dataset (Supplementary Table IV). Three SNPs showed association with *P* < 0.01: rs10501087 (*BDNFOS*), rs2179129 (*ZNRF3-KREMEN1*), and rs4846565 (*LYPLAL1*). The aforementioned six EDs and obesity-associated SNPs were selected for *de novo* replication in the TEENAGE cohort. Of the six SNPs taken forward to de novo genotyping, none showed association with *P* < 0.05 in the TEENAGE cohort. Four SNPs had the same direction of effect in the TEENAGE cohort as in the discovery dataset (binomial *P*-value = 0.687). Meta-analysis across the TwinsUK and TEENAGE datasets resulted in two SNPs with *P* < 10^−4^ in DT analysis (Supplementary Table V).

## DISCUSSION

In this large-scale analysis of six ED phenotypes, we analyzed a total of 2,698 individuals for DT, 2,680 for BD, 2,789 for Bulimia, 1,360 for OCPD, 2,773 for Breakfast Skipping, and 2,967 for WF across a GWAS discovery dataset and two replication cohorts of European ancestry. We followed-up signals from various strata in a two stage replication but found no signals reaching genome-wide significance.

The power of the study varied across the six investigated ED phenotypes. Given the fact that the three analyzed datasets comprise individuals that are comparable to the age-matched general population, analyses of dichotomized ED phenotypes resulted with a relatively limited number of individuals who scored in the pathological range and were analyzed as cases (Tables I and II). In general, meta-analysis had 80% power to detect large to modest effects at common loci at the genome-wide significance level. We did not have power to detect small effect sizes at common loci, especially for dichotomized traits, and have not examined the effect of low frequency and rare variants on ED phenotypes.

The genetic architecture of EDs is still not well-understood [Helder and Collier, 2011] but it is considered to result from environmental factors acting together with common and rare genetic variants [Moore and Williams, 2009]. Usually, common genetic risk variants have relatively low penetrance and are responsible for a small increase in disease risk [Schork et al., 2009]. This is consistent with our observations, where genetic variants with the greatest evidence of association (Table III) show relatively small-to-modest effect sizes that could not be detected at genome-wide significance level by the investigated sample size. Moreover, Uher suggested that gene–environment interactions and cumulative effects of rare genetic variants have a major role in defining genetic susceptibility to psychiatric disorders [Uher, 2009]. Visscher et al., [2011], however, argue that even though most variants underlying susceptibility for psychiatric disorders are expected to be rare, most variation in the population is expected to be due to common variants. Our study was not powered to detect rare variants with small to modest effect sizes, which were also underrepresented on the genotyping platforms used in the present study.

In this study, we examined ED-related symptoms, behaviors, and traits which are related to ED diagnoses. It is generally thought that ED-related symptoms, behaviors, and traits have a similar genetic background to EDs but may have less complex genetic architecture (i.e., fewer genes may be involved), similarly as endophenotypes, although the hypothesis that endophenotypes are less complex remains unproven [Gottesman and Gould, 2003; Morris et al., 2010]. Perhaps more importantly, EDs are longitudinally unstable diagnoses, creating obvious difficulties for diagnostic genetic studies, whereas symptoms, behaviors, and trait measures are present during the lifetime of individuals and may be more stable phenotypes [Helder and Collier, 2011]. In addition, some symptoms, behaviors, and traits may be useful as trans-diagnostic traits that can offer insight in different classes of psychiatric diseases [Bulik et al., 2007]. For example, DT is considered to be a symptom seen across EDs because it is a composite measure of weight concern incorporating BD, dieting, and importance of weight [Bulik et al., 2007]. Therefore, genetic analysis of these variables can offer advantages in detecting disease susceptibility loci and help in the dissection of psychiatric diseases and biological mechanisms contributing to them [Bulik et al., 2007]. On the other hand, using the Bulimia scale as a binary trait is not straightforward and it is unclear whether its dichotomization truly captured a bulimia phenotype—especially as very few individuals would meet the diagnostic threshold for bulimia in the present study. It is possible that the dimensions we have chosen have insufficient variability on the extreme end to be meaningful for genetic studies of EDs, as they can all be regarded as normative traits. Thus the EDI-2, which was developed for use in a clinical setting, may capture something different in the general population, as would occur in the hypothetical situation where EDs were a rare genetic variant disorder. On the other hand, if EDs had a significant contribution from common, low risk variants, population study may reveal important information on genetic risk. It is also important to keep in mind that risk allele effect sizes for symptoms, behaviors, and traits, even when they are identified as endophenotypes, are comparable, and not larger than those observed for clinical diseases arguing against the belief that the genetic basis of endophenotypes will be easier to analyze [Flint and Munafo, 2007]. Our results go in line with this statement since the genetic variants with the greatest evidence of association (Table III) have relatively small-to-modest effect sizes which are consistent with general expectations of effect sizes for psychiatric traits.

Genome-wide data for the discovery TwinsUK dataset were available for female–female twin pairs only, therefore, analyses were performed only in females. Replication studies consisted of both males and females. We analyzed both genders in replication sets to increase the sample size and the power of the study. Two previously published studies analyzed similarities and differences between men and women for a set of ED risk factors and symptom measures, including EDI, and found that the majority of these scores measure similar constructs in both genders but men generally score lower than women and provide less reliable results [Boerner et al., 2004; Spillane et al., 2004]. This may have slight implications on the power of our study, since we used the same thresholds for binary traits in both sexes, but this is unlikely to have a major effect on study results and conclusions. The twinsUK dataset differs from replication studies by the mean age of individuals, that is, TwinsUK consist of middle aged females (mean age 58.2 years), whereas FinnTwin16 is comprised of individuals in their 20s (mean age 24.96 years) and TEENAGE of adolescents (mean age 15.27). In addition, mean values for Bulimia and OCPD traits were higher in TEENAGE samples that those observed in TwinsUK and FinnTwin16, whereas Breakfast Skipping was increased in both replication cohorts when compared to TwinsUK. Several studies have already implicated the existence of socio-cultural differences in dimensions measured by EDI in European populations. A cross-cultural comparison of the EDI measures showed that non-Western healthy participants usually score higher than Western participants on EDI subscales and that the age is a risk factor for individuals that are already diagnosed for EDs but that age only decreases the likelihood of developing EDs in the general population [Podar and Allik, 2009]. Another study pointed to age-dependent North-South variation of EDI scores where individuals from the South score higher than individuals from the North [Waldherr et al., 2008]. This is consistent with our observation where Greek TEENAGE individuals have on average higher scores for the majority of investigated traits (Tables I and II). A limiting factor of our study is that we have not adjusted our analyses for age or sex (since the discovery dataset consisted of females only) and that even though the investigated phenotypes were assessed in a normal population, it is possible that cases were inadvertently included.

Our study detected eight SNPs with *P* < 10^−5^ (Table III). Some of these suggestively associated variants have already been associated with several psychiatric and other ED-related risk factors. For example, SNP rs7624327, the most significantly associated variant in the Bulimia analysis, lies between the *CCNL1* and *LEKR1* genes. Variants near *CCNL1*/*LEKR1* have already been associated with fetal growth and birth weight [Freathy et al., 2010]. The birth weight lowering allele near *CCNL1*/*LEKR1* has also shown association with elevated insulin release following an oral glucose stimulation [Andersson et al., 2011]. *CCNL1* has also been implicated with several types of cancer including breast cancer [Peng et al., 2011]. SNP rs1898111, that showed suggestive association with OCPD, lies within *SEMA6D*. SEMA6D is involved in neural wiring of the central nervous system [Leslie et al., 2011]. It also belongs to a large semaphorin family, many members of which act as inhibitors or chemorepellents in neuronal development [Qu et al., 2002]. The other suggestively associated OCPD SNP, rs10519201, resides in the *SHC4* gene. This gene showed female-specific association with another psychiatric illness, major depressive disorder, in a Dutch sample set [Aragam et al., 2011]. This is in line with our finding since all individuals in the study that is driving the signal, TwinsUK, are females. Gender-specific mechanisms may have a role in the development of psychiatric disorders and genes may act broadly across various psychiatric traits. The third OCPD associated SNP, rs1557305, lies ∼260 kb from the *DLGAP1* gene that has been associated with latitude-driven genetic adaptation related to schizophrenia and vitamin D metabolism [Amato et al., 2010]. Finally, the WF suggestively associated variant rs218361 near the *TRPS1* gene was picked up by extensive database searches as a candidate gene for the sleep disorder narcolepsy [Shimada et al., 2010].

One of the aims of our study was to examine if SNPs that were previously implicated/associated by candidate gene and GWAS studies with EDs and obesity are also associated with the six ED phenotypes investigated in our study. Analysis of the TwinsUK discovery dataset resulted in six SNPs with *P* < 0.01 that were further followed up in the TEENAGE cohort. Meta analysis of discovery and replication cohorts resulted in two SNPs with *P* < 10^−4^ in DT analysis: rs6265 within *BDNF*, previously associated with AN, and rs10501087 within *BDNFOS*, previously associated with BMI (Supplementary Table V). Both of these SNPs are in LD with each other based on the 1,000 genomes project (r^2^ = 0.817). The same SNP in the *BDNF* gene was previously associated with smoking initiation [The Tobacco and Genetics Consortium, 2010]. Smoking status can be a confounding factor in genetic association analyses of EDs since it was shown to be associated with disordered eating symptoms, especially among female adolescents [Potter et al., 2004]. The confounding effect in the suggested association is possible but it is less likely to have big effect since the study that is driving the association, TwinsUK, is composed of females with age range from 16 to 85 and with a lower rate of tobacco use. We have not adjusted our analyses for smoking status.

In this large-scale GWAS analysis of six ED phenotypes, we did not identify signals reaching genome-wide significance. Several variants that have previously been associated with psychiatric disorders showed suggestive evidence for association with the ED phenotypes investigated here. The examination of variants across the entire allele frequency spectrum accompanied with systematic gene–environment interaction analysis will be necessary to thoroughly examine the genetic basis of ED phenotypes. Importantly, large-scale collaborative studies will be required to achieve the necessary power.
