# Supplementary material for: Genome-Wide Association Analysis of Eating Disorder-Related Symptoms, Behaviors, and Personality Traits
Source: Am J Med Genet B Neuropsychiatr Genet. 2012 Aug 22;159B(7):803–11. doi: 10.1002/ajmg.b.32087 (PMC3494378; doi:10.1002/ajmg.b.32087)
Supplement: Supplementary file 2 [file ajmg0159B-0803-SD2.doc]

Supplementary Table 2: Variance accounted for by tagged SNPs in the discovery TwinsUK dataset

| **Phenotype** | **Type** | **Sample** | **Prevalence** | **Variance explained** | **SE** | **P** |
| --- | --- | --- | --- | --- | --- | --- |
| DT | qt | 1206 | - | 0.27 | 0.28 | 0.17 |
| BD | qt | 1191 | - | 0 | 0.28 | 0.5 |
| BULIMIA | bn | 1262 | 0.26 | 0 | 0.49 | 0.5 |
| OCPD | bn | 547 | 0.3 | 0.21 | 1.03 | 0.42 |
| BREAKFAST SKIPPING | bn | 1250 | 0.19 | 0 | 0.53 | 0.5 |
| WF | bn | 1604 | 0.26 | 0 | 0.37 | 0.5 |

Phenotypes: Drive For Thinness (DT), Body Dissatisfaction (BD), Childhood Obsessive Compulsive Personality Disorder (OCPD), Weight Fluctuation (WF); qt—quantitative trait; bn—binary trait; SE—standard error; P value from comparison with the null model where the genotyped SNPs account for no variance in the trait

*Variance accounted for by tagged SNPs.* Supplementary table 2 shows the proportion of the phenotypic variance accounted for by the SNPs tagged by the array. Reducing the sample to unrelated individuals and the relatively small number of overlapping SNPs limited the power of this analysis, so standard errors are large and none of the variance components are statistically significant compared to the null model where tagged SNPs account for none of the phenotypic variance. However, the point estimate for DT is 27% and the point estimate for OCPD is 21%. Compared to the twin heritability estimates of 57% and 81% respectively, these results suggest that the array accounts for around half of the genetic variance attributable to DT, and a quarter of the genetic variance attributable to OCPD (although the large standard errors mean that these estimates should be treated with caution).
