# Supplementary material for: Genome-Wide Association Analysis of Eating Disorder-Related Symptoms, Behaviors, and Personality Traits
Source: Am J Med Genet B Neuropsychiatr Genet. 2012 Aug 22;159B(7):803–11. doi: 10.1002/ajmg.b.32087 (PMC3494378; doi:10.1002/ajmg.b.32087)
Supplement: Supplementary file 6 [file ajmg0159B-0803-SD6.doc]

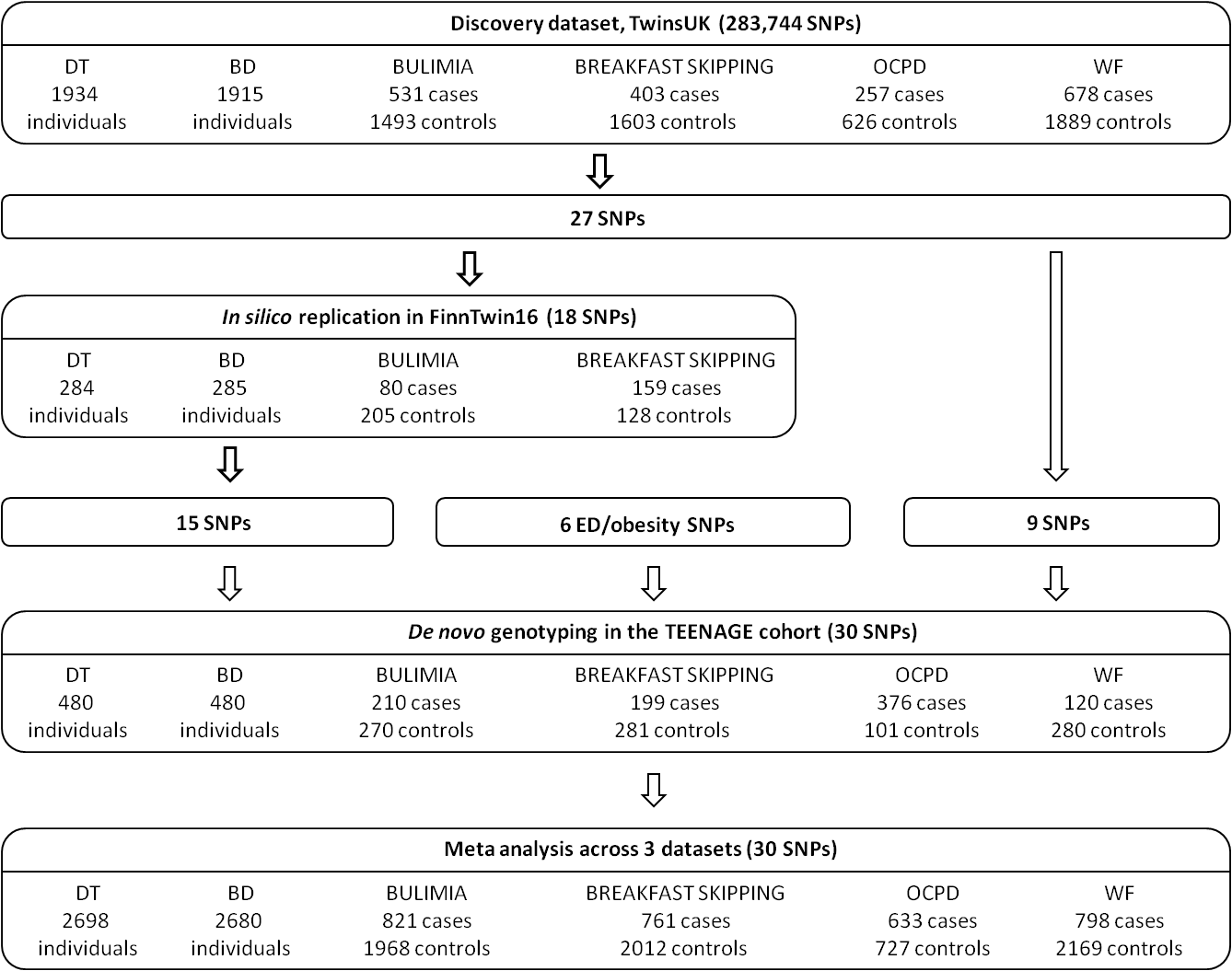


Supplementary Figure 1. Study design. Each step includes non-overlapping, independent datasets. Single nucleotide polymorphism (SNP), Drive For Thinness (DT), Body Dissatisfaction (BD), Childhood Obsessive Compulsive Personality Disorder (OCPD) trait, Weight Fluctuation (WF), Eating disorders (ED).
