# Supplementary material for: Genome-Wide Association Analysis of Eating Disorder-Related Symptoms, Behaviors, and Personality Traits
Source: Am J Med Genet B Neuropsychiatr Genet. 2012 Aug 22;159B(7):803–11. doi: 10.1002/ajmg.b.32087 (PMC3494378; doi:10.1002/ajmg.b.32087)
Supplement: Supplementary file 7 [file ajmg0159B-0803-SD7.doc]

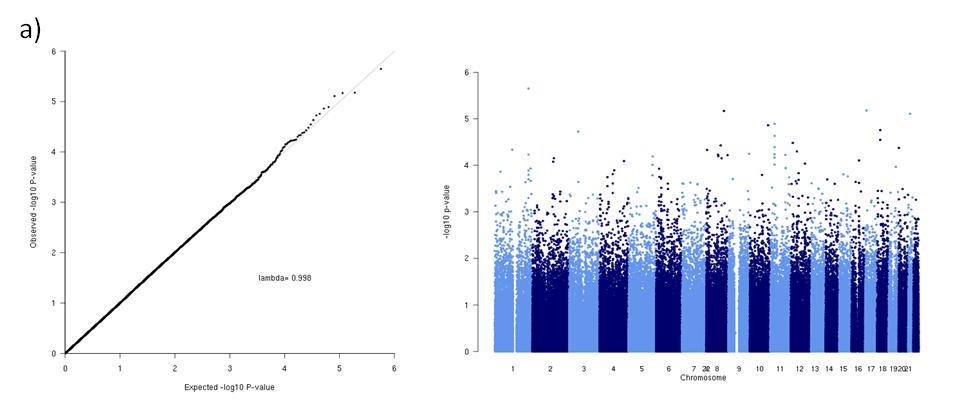


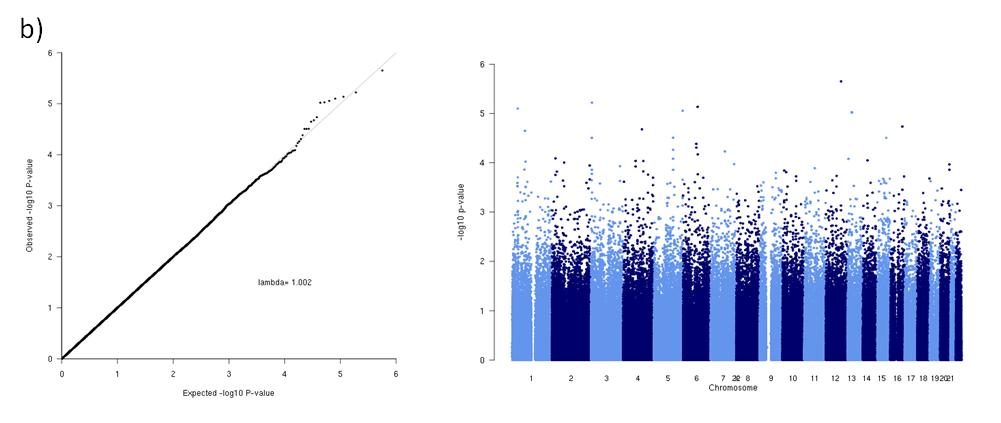


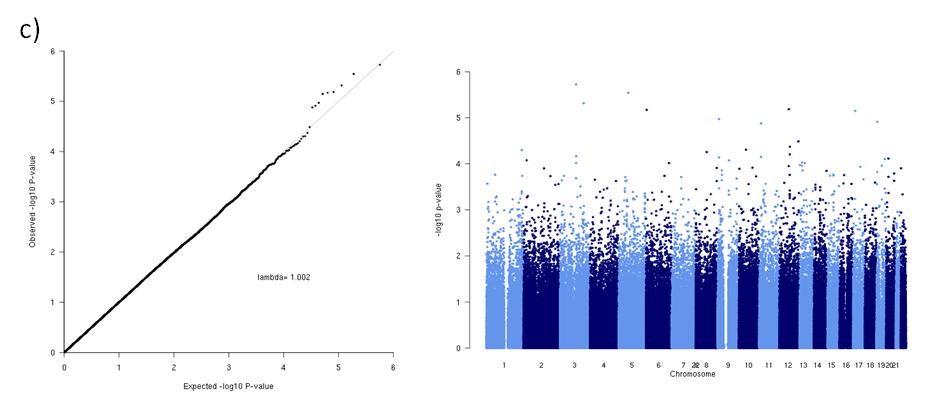


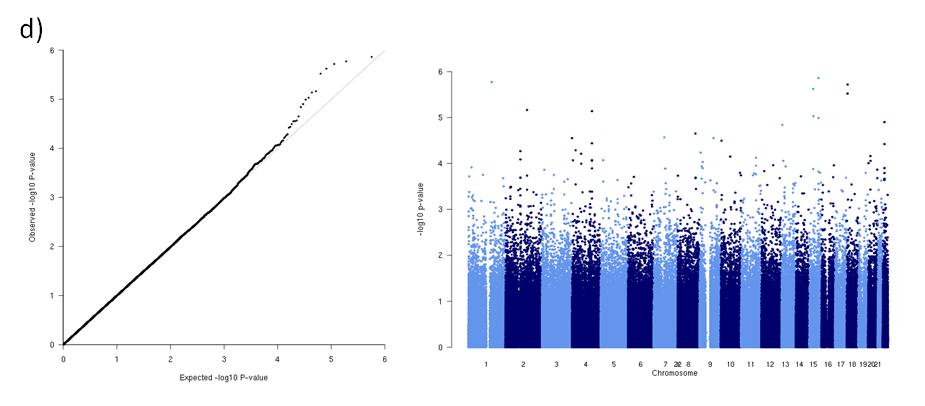


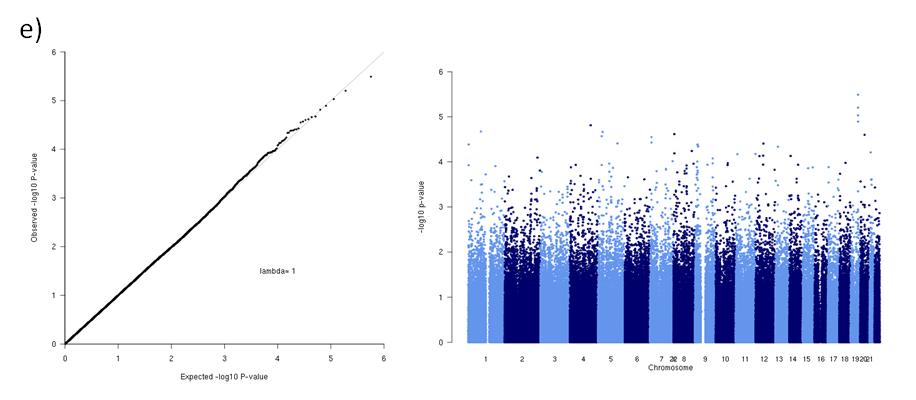


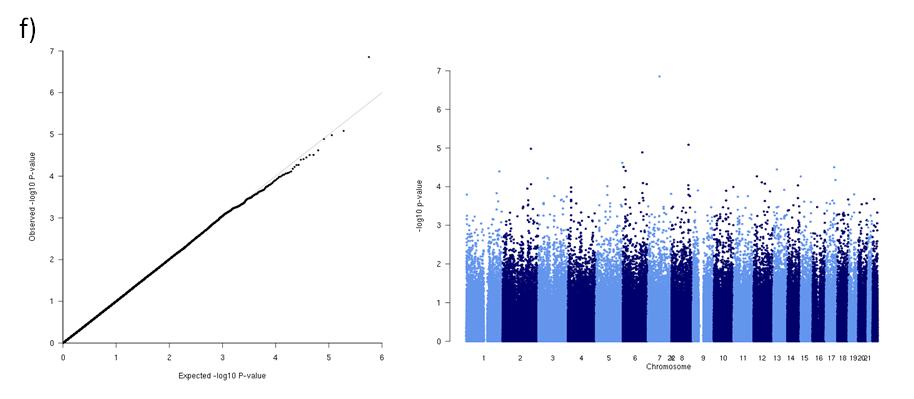


Supplementary Figure 2. Manhattan and QQ plots based on meta-analyses results of the discovery dataset: a) Drive For Thinness (DT); b) Body Dissatisfaction (BD); c) Bulimia; d) Childhood Obsessive Compulsive Personality Disorder (OCPD); e) Breakfast Skipping; f) Weight Fluctuation (WF)
