# Supplementary material for: Genome-Wide Association Analysis of Eating Disorder-Related Symptoms, Behaviors, and Personality Traits
Source: Am J Med Genet B Neuropsychiatr Genet. 2012 Aug 22;159B(7):803–11. doi: 10.1002/ajmg.b.32087 (PMC3494378; doi:10.1002/ajmg.b.32087)
Supplement: Supplementary file 8 [file ajmg0159B-0803-SD8.doc]

**supplementaRY MATERIAL**

**Materials and methods**

***TwinsUK subjects***

These twins are part of a predominantly female volunteer register (Spector and Williams 2006) that has been built up over many years through national media campaigns and from other twin registers. Twins have not been selected by any phenotype and it was shown that they are comparable to the age-matched general population for a wide range of medical and behavioural variables (Andrew et al. 2001). The study was approved by the St Thomas’ Hospital research ethics committee, and all twins in the study provided informed consent. Only female-female monozygotic (MZ) and dizygotic (DZ) twin pairs were included in the present study since the genotype data was only available for females. More information on twin characteristics and phenotypes can be found at the website twinsuk.ac.uk/.

***Genome-wide genotyping and quality control (QC) procedures of the TwinsUK dataset***

*Genotyping*. Genotyping of the TwinsUK dataset was done with a combination of Illumina arrays (HumanHap300, HumanHap610Q, 1M-Duo and 1.2MDuo 1M) (Richards et al. 2008; Soranzo et al. 2009). We pooled the normalised intensity data (Kermani 2005) for each of the three arrays separately (with 1M-Duo and 1.2MDuo 1M pooled together). For each dataset we used the Illuminus calling algorithm (Teo et al. 2007) to assign genotypes in the pooled data. No calls were assigned if an individual's most likely genotype was called with a posterior probability threshold less than 0.95. Validation of pooling was achieved via a visual inspection of 100 random, shared SNPs for overt batch effects. Finally, intensity cluster plots of significant SNPs were visually inspected for over-dispersion biased no calling, and/or erroneous genotype assignment. SNPs exhibiting any of these characteristics were discarded.

*Data QC*. We applied similar exclusion criteria to each of the three datasets separately. Samples: Exclusion criteria were: (i) sample call rate <98%, (ii) heterozygosity across all SNPs ≥2 standard deviations (s.d.) from the sample mean; (iii) evidence of non-European ancestry as assessed by PCA comparison with HapMap3 populations; (iv) observed pairwise IBD probabilities suggestive of sample identity errors. We corrected misclassified monozygotic and dizygotic twins based on IBD probabilities. SNPs: Exclusion criteria were (i) Hardy-Weinberg p-value<10^−6^, assessed in a set of unrelated samples; (ii) MAF<1%, assessed in a set of unrelated samples; (iii) SNP call rate <97% (SNPs with MAF≥5%) or < 99% (for 1% ≤ MAF < 5%). Alleles of all three datasets were aligned to HapMap2 or HapMap3 forward strand alleles.

*Data merge*. Prior to merging, we performed pairwise comparison among the three datasets and further excluded SNPs and samples to avoid spurious genotyping effects, identified as follows: (i) concordance at duplicate samples <1%; (ii) concordance at duplicate SNPs <1%; (iii) visual inspection of QQ plots for logistic regression applied to all pairwise dataset comparisons; (iv) Hardy-Weinberg p-value<10^−6^, assessed in a set of unrelated samples; (v) observed pairwise IBD probabilities suggestive of sample identity errors. We then merged the three datasets, keeping individuals typed at the largest number of SNPs when an individual was typed at two different arrays. The merged dataset consists of 5,654 individuals (2,040 from the HumanHap300, 3,461 from the HumanHap610Q and 153 from the HumanHap1M and 1.M arrays) and up to 874,733 SNPs depending on the dataset (HumanHap300: 303,940, HumanHap610Q: 553,487, HumanHap1M and 1.M: 874,733).

***Self-report questionnaires in TwinsUK***

*The Eating Disorders Inventory 2* (EDI-2) (Garner 1991) is a standardised self-report assessment that consists of several subscales. We examined three subscales that assess DT, BN and BD. The EDI-2 responses were scored from 1-6 (always, usually, often, sometimes, rarely, never). The BD subscale consisted of 10, DT of 7 and Bulimia of 8 items. BD scores showed normal distribution and 1/squared root transformation was applied to normalise the distribution of DT scores. BD and DT were analysed as quantitative traits. Bulimia scores could not be transformed to normality hence Bulimia was analysed as a binary trait with 12 set as an arbitrary cut-off point beyond which individuals have more tendencies to develop bulimia. We excluded two questions from BD and one question from Bulimia subscales to make these traits comparable with traits measured by replication studies (FinnTwin16 and TEENAGE cohorts).

*Childhood Retrospective Perfectionism Questionnaire* (CHIRP) (Southgate et al. 2008) is a short retrospective self-report measure of childhood OCPD traits. Childhood is defined up to 12 years of age to ensure that these traits are assessed before the ED onset (Fairburn and Harrison 2003). The questionnaire consists of 19 questions with yes/no answers, where higher score represents greater obsessive-compulsive personality tendencies in childhood. The distribution of scores in the TwinsUK sample was skewed to the left hence OCPD was analysed as a binary trait. The cut-off point for differentiation of individuals who score in the pathological range was 6.

*Weight fluctuation* (WF) was measured by the Hermann and Polivy Restraint Scale questionnaire (Heatherton et al. 1991). It consists of 4 questions with a score range from 0-16. The distribution of scores was skewed hence WF was analysed as a binary trait. The cut-off point for differentiation of individuals who score in the pathological range was 9.

*Breakfast Skipping* was assessed by a 1-item question (5-point Likert scale) "How often do you eat breakfast (e.g. bread, toast, milk) at the start of the day?" Five alternative responses were listed as every morning, 5-6 days/week, 3-4 days/week, 1-2 days/week and less than 1 day/week. Higher score represents higher drive for Breakfast Skipping (Keski-Rahkonen et al. 2004). Breakfast Skipping was analysed as a binary trait since most of the TwinsUK subjects had breakfast everyday and were therefore divided into two groups, those who have breakfast everyday and those who skip breakfast once or more per week.

***Association analysis***

We tested 283,744 directly typed overlapping SNPs for association with the six ED phenotypes of interest using GenABEL (Aulchenko et al. 2007). All analyses were adjusted for family relatedness i.e. based on the trait and kinship matrix, a linear mixed (polygenic) model was used to estimate residuals of the trait and the inverse of the variance-covariance matrix. These residuals were then used in analysis using a score test for association between SNPs and the trait of interest.

***Estimating the total variance accounted for by tagged SNPs in TwinsUK***

We estimated the proportion of the phenotypic variation accounted for by the 283,744 overlapping SNPs in the TwinsUK sample using the method described by Yang et al. (Yang et al. 2010) and the GCTA software (http://gump.qimr.edu.au/gcta/). This approach is complementary to the twin analysis reported earlier: whereas the twin analysis calculates heritability, taking into account all forms of genetic variation, GCTA takes into account only variants tagged by the genotyped SNPs (Visscher et al. 2010). This estimate can be compared to the heritability estimate from twin analysis to estimate the “missing” heritability that remains to be accounted for by variants not tagged by the array. To avoid inflation of the GCTA estimate by shared environmental influences or untagged pedigree information, for this analysis we excluded one member from each pair of participants who were more closely related than an arbitrary threshold of 0.025 (including one member of each twin pair). In each case we retained the individual with more genotype information, leaving a sample of 1836 individuals.

***In silico replication: FinnTwin16 dataset***

The twins from the FinnTwin16 cohort (Kaprio et al. 2002) were sent self-report questionnaires (fourth wave of data collection) between the ages of 22 and 28. A total of 2881 participants responded to the wave 4 questionnaire (response rate 90%), the mean age of respondents was 24.4 years (SD=0.9). Female controls were ascertained from the FinnTwin16 cohort members with GWAS data such that one individual from each twin pair was included; twin sisters of AN cases were excluded. Study protocols were reviewed and approved in advance by Institutional Review Boards/Research Ethical committees at the University of Helsinki and Hospital Distric of Northern Savo. All participants provided written informed consent.

***Genome-wide genotyping and QC procedures of the FinnTwin16 dataset***

*Genotyping and QC procedure:* DNA was extracted from blood samples or Oragene saliva kits using standard procedures and genotyped using Illumina 670-Quad Custom chips at two different timepoints. The following QC criteria were applied: a) minor allele frequency of markers had to exceed 1%; b) genotyping success rate per marker had to exceed 95%; c) individuals were removed if the genotyping success rate per individual was under 95%. Using one individual per family, we excluded markers based on the HWE test (p <= 1x10^-6^). Sex was checked and individuals were removed based on the heterozygosity test for inbreeding (F>0.05). Finally, multidimensional scaling showed that clean dataset forms one cluster, with no deviating points/clusters (individuals). This is what we would expect to see in a dataset consisting of unrelated individuals of Finnish origin. The final dataset analyzed in the present study included the target SNPs for 291 individuals who had completed 4 ED-related traits questionnaires (DT, BD, Bulimia and Breakfast Skipping).

***De novo replication:*** ***TEENAGE cohort***

ED-related questionnaires were translated to Greek and back-translated to English in order to check for accuracy of the translation. ED phenotypes were measured by self-report questionnaires by 480 unrelated subjects of the TEENAGE cohort (TEENs of Attica: Genes & Environment) study. The TEENAGE target population was comprised of adolescent students attending all three classes of public secondary schools in the Attica region of Greece. The research study was approved by the Institutional Review Board of Harokopio University and the Greek Ministry of Education, Lifelong Learning, and Religious Affairs. Prior to recruitment, registered students in the participating secondary schools as well as their parents/ guardians received written information about the study design and were assured of the voluntary nature of the study and the confidentiality of the data. Participating students provided their assent along with a written voluntary consent signed from their parents/guardians. Participants were asked to complete the ED-related questionnaires 1-2 years after their recruitment to the study.

***De novo genotyping, QC procedures and association analysis in the TEENAGE dataset***

Genotyping of 30 prioritised SNPs was performed using the iPLEX™ Gold Assay (Sequenom® Inc.). Assays for all SNPs were designed using the eXTEND suite and MassARRAY Assay Design software version 3.1 (Sequenom® Inc.). Assays for one SNP (rs2179129) could not be designed, instead we genotyped its proxy (rs2294239). Amplification was performed in a total volume of 5µL containing ~0.06-0.4ng genomic DNA, 100nM of each PCR primer, 500µM of each dNTP, 1.25 x PCR buffer (Qiagen), 1.625mM MgCl₂ and 1U HotStarTaq® (Qiagen). Reactions were heated to 94 °C for 15 min followed by 45 cycles at 94 °C for 20 s, 56 °C for 30 s and 72 °C for 1 min, then a final extension at 72 °C for 3 min. Unincorporated dNTPs were SAP digested prior to iPLEX™ Gold allele specific extension with mass-modified ddNTPs using an iPLEX Gold reagent kit (Sequenom® Inc.). SAP digestion and extension were performed according to the manufacturer’s instructions with reaction extension primer concentrations adjusted to between 0.7-1.8µM, dependent upon primer mass. Extension products were desalted and dispensed onto a SpectroCHIP using a MassARRAYNanodispenser prior to MALDI-TOF analysis with a MassARRAY Analyzer Compact mass spectrometer. Genotypes were automatically assigned and manually confirmed using MassARRAYTyperAnalyzer software version 4.0 (Sequenom® Inc.).

We applied the following sample and SNP QC exclusions for *de novo* genotype data: mismatch sex, samples call rate <98% and HWE p-value<0.0001. Average assay call rate was 0.997. Overall, 480 samples and all 30 SNPs passed QC criteria. An additive linear or logistic, where appropriate, regression model was applied using Plink (Purcell et al. 2007).

**References**

Andrew T, Hart DJ, Snieder H, de Lange M, Spector TD, MacGregor AJ. 2001. Are twins and singletons comparable? A study of disease-related and lifestyle characteristics in adult women. Twin Res 4: 464-77

Aulchenko YS, Ripke S, Isaacs A, van Duijn CM. 2007. GenABEL: an R library for genome-wide association analysis. Bioinformatics 23: 1294-6

Fairburn CG, Harrison PJ. 2003. Eating disorders. Lancet 361: 407-16

Garner DM. 1991. Eating Disorder Inventory 2, professional manual. Odessa, FL: Psychological Assessment Resources

Heatherton TF, Polivy J, Herman CP. 1991. Restraint, weight loss, and variability of body weight. J Abnorm Psychol 100: 78-83

Kaprio J, Pulkkinen L, Rose RJ. 2002. Genetic and environmental factors in health-related behaviors: studies on Finnish twins and twin families. Twin Res 5: 366-71

Kermani B. 2005. Artificial intelligence and global normalization methods for genotyping.

Keski-Rahkonen A, Viken RJ, Kaprio J, Rissanen A, Rose RJ. 2004. Genetic and environmental factors in breakfast eating patterns. Behav Genet 34: 503-14

Purcell S, Neale B, Todd-Brown K, Thomas L, Ferreira MA, Bender D, Maller J, Sklar P, de Bakker PI, Daly MJ, Sham PC. 2007. PLINK: a tool set for whole-genome association and population-based linkage analyses. Am J Hum Genet 81: 559-75

Richards JB, Rivadeneira F, Inouye M, Pastinen TM, Soranzo N, Wilson SG, Andrew T, Falchi M, Gwilliam R, Ahmadi KR, Valdes AM, Arp P, Whittaker P, Verlaan DJ, Jhamai M, Kumanduri V, Moorhouse M, van Meurs JB, Hofman A, Pols HA, Hart D, Zhai G, Kato BS, Mullin BH, Zhang F, Deloukas P, Uitterlinden AG, Spector TD. 2008. Bone mineral density, osteoporosis, and osteoporotic fractures: a genome-wide association study. Lancet 371: 1505-12

Soranzo N, Rivadeneira F, Chinappen-Horsley U, Malkina I, Richards JB, Hammond N, Stolk L, Nica A, Inouye M, Hofman A, Stephens J, Wheeler E, Arp P, Gwilliam R, Jhamai PM, Potter S, Chaney A, Ghori MJ, Ravindrarajah R, Ermakov S, Estrada K, Pols HA, Williams FM, McArdle WL, van Meurs JB, Loos RJ, Dermitzakis ET, Ahmadi KR, Hart DJ, Ouwehand WH, Wareham NJ, Barroso I, Sandhu MS, Strachan DP, Livshits G, Spector TD, Uitterlinden AG, Deloukas P (2009) Meta-analysis of genome-wide scans for human adult stature identifies novel Loci and associations with measures of skeletal frame size. PLoS Genet 5: e1000445

Southgate L, Tchanturia K, Collier D, Treasure J. 2008. The development of the childhood retrospective perfectionism questionnaire (CHIRP) in an eating disorder sample. Eur Eat Disord Rev 16: 451-62

Spector TD, Williams FM. 2006. The UK Adult Twin Registry (TwinsUK). Twin Res Hum Genet 9: 899-906

Teo YY, Inouye M, Small KS, Gwilliam R, Deloukas P, Kwiatkowski DP, Clark TG. 2007. A genotype calling algorithm for the Illumina BeadArray platform. Bioinformatics 23: 2741-6

Visscher PM, Yang J, Goddard ME. 2010. A commentary on 'common SNPs explain a large proportion of the heritability for human height' by Yang et al. (2010). Twin Res Hum Genet 13: 517-24

Yang J, Benyamin B, McEvoy BP, Gordon S, Henders AK, Nyholt DR, Madden PA, Heath AC, Martin NG, Montgomery GW, Goddard ME, Visscher PM. 2010. Common SNPs explain a large proportion of the heritability for human height. Nat Genet 42: 565-9
